# Prophylactic consequences of sodium salicylate nanoparticles in cisplatin-mediated hepatotoxicity

**DOI:** 10.1038/s41598-023-35916-9

**Published:** 2023-06-21

**Authors:** Maha Alkhalaf, Nadia A. Mohamed, Safinaz E. El-Toukhy

**Affiliations:** 1 Biochemistry Department, Faculty of Science, University of Jeddah, Jeddah, Saudi Arabia; 2 Medical Biochemistry Department, National Research Centre, Cairo, Egypt

**Keywords:** Biochemistry, Cell biology, Chemical biology, Molecular biology

## Abstract

Unintended side effects linked to the antineoplastic drug cisplatin are a major drawback in its clinical application. The underlying source of these side effects include the generation of reactive oxygen species which are toxic and damaging to tissues and organs. In the present study the anti-inflammatory and antioxidant potential of sodium salicylate was assessed against cisplatin-induced hepatotoxicity in albino rats. Sodium salicylate was used as a model drug and loading into hollow structured porous silica using ultrasound-assisted sol–gel method to produce a nanoemulsion. Transmission Electron Microscopy and Dynamic Light scattering analysis were employed to assess the structural properties and stability of this model. Liver function was assessed by measuring biomarkers including ALT, AST & GGT and oxidant/antioxidant markers including MDA, NO, PON, GSH, MCP1 & AVP in serum or liver tissue. Additionally, blood leukocyte DNA damage was evaluated. Cisplatin significantly altered the normal levels of all biomarkers confirming its hepatotoxic effects. In contrast, treatment with sodium salicylate-loaded silica nanoemulsion significantly restored the levels of these markers. The finding suggests the protective effects of this model drug in preventing cisplatin-induced hepatotoxicity, and therefore may have implications in attenuating cisplatin-induced hepatotoxicity.

## Introduction

Cisplatin, also known as cisplatinum or cis-diamminedichloroplatinum (II), is a chemotherapeutic drug administered alone or in combination with another drug. Cisplatin is widely utilized in the treatment of number of cancer types including ovarian, bladder, testicular, and lung^1^. Due to its unique clinical advantages, cisplatin has been the choice of drug in the cancer treatment. Its mechanism of action includes induction of apoptosis by crosslinking the purine bases of DNA, which results in DNA damage^2^. However, cisplatin treatment is associated with a number of side effects including renal dysfunction^3–5^, hepatotoxicity^5,6^, nausea and vomiting^7^. Cisplatin was found to accumulate in kidneys due to basolateral organic cation transporters which contributes to its nephrotoxicity^8^. Based on studies in animals and cells, it is hypothesized that nephrotoxicity induced by cisplatin is mediated by the inflammation, oxidative stress, and cell death^9^.

Hepatotoxicity is not regarded as a dose-limiting toxicity for cisplatin but can occur when the drug is administered at high doses, proving an obstacle in its continued use^10^. Adverse drug reactions affecting the liver function remained a major challenge in the use of cisplatin. Acute liver failure is the most severe clinical consequence leading to drug-mediated fatality^11^.

Salicylates are commonly used in the treatment of inflammatory diseases such as rheumatoid arthritis and rheumatic fever. The anti-inflammatory actions of non-steroidal anti-inflammatory drugs including salicylates have been attributed to their ability to inhibit prostaglandin synthesis and cyclooxygenase activity. Salicylates at high doses also inhibit the IKB kinase β (IKK) activity, resulting in the attenuation of nuclear factor kappa B (NF-jB) transcriptional activity^12^. Moreover, salicyalates abrogated cisplatin-induced leukocyte infiltration, TNF-α production and kidney dysfunction. Importantly, mitochondria can be a model for studies on anticancer drug-induced hepatotoxicity^13^. Antitumor activities of cisplatin and most other platinum compounds are mediated through signal transduction and mitochondrial pathways that lead to the induction of apoptosis. The mechanism of cisplatin action involves its reaction with nucleophilic sites of DNA, resulting in the formation of monoadducts and intra and interstrand cross-links^14^. Additional mechanisms in the cisplatin induced toxicity are also proposed such as the generation of reactive oxygen species^15^. Inside the cell cisplatin disturbs the redox status either by lowering the endogenous antioxidants, NADPH and GSH or damaging the inner membrane of mitochondrial resulting in influx of reactive nitrogen and oxygen species^16^. These reactive oxygen and nitrogen species in turn induce apoptosis. Higher doses of cisplatin are more effective in resolving cancer but are linked to unintended non-tumor cell damage. Hence, drug combinations either synergistic or complementary in their actions are suggested to be an important strategy to avoid the collateral cell damage^17^.

Due to the complexity of tumor microenvironment composition, the penetration ability of nanomedicine into tumors has become an important factor determining the therapeutic effect. With the development of nanomedicine research, the visual monitoring of tumor disease development and treatment process has become an important link to improve the quality of treatment^18^. Doping substances into silica matrix is one of the most common methods for promoting biodegradation, which reduces the densification of silica matrix and speeds up the hydrolysis of silica nanomaterials^19^.

Surface modification of nanomedicine to improve their adaptability to the tumor microenvironment is one of the most effective methods to realize the deep penetration of nanomedicine. Therefore, nanomedicines, which circulate in the bloodstream with a negative surface charge but can be reversed to a positive surface charge when they reach the tumor site, produce better tumor penetration^20^. For example, deep penetration in tumor tissues was obtained with PEOz-functionalized PDA-Gd-modified nanoparticles for chemo/photothermal synergistic therapy^21^.

Oxidative stress is an integral part of cisplatin-induced hepatotoxicity. Oxidative stress caused due to an imbalance in reactive oxygen species and antioxidants. This imbalance could be attributed to an increase in free radicals or a decrease in antioxidants. Several studies have demonstrated that an increase in oxidative stress affects malondialdehyde (MDA) levels, and a decrease in antioxidants such as glutathione (GSH), and catalase (CAT), glutathione peroxidase (GSH-Px) and superoxide dismutase (SOD) activities in liver tissue in response to cisplatin treatment^10,22^. Therefore, blunting oxidative stress is an important aspect of hepatotoxicity induced by cisplatin^23^.

Monocyte chemoattractant protein 1 (MCP-1) is an important player in the regulation of monocyte/macrophage function which underlies chronic inflammatory disease etiologies. MCP-1 aids in directing the flow of immune cells to the site of tissue damage^24^. Additionally, MCP-1 is shown to participate in metabolic diseases such as obesity-related insulin resistance, diabetes and hepatic steatosis^25^.

The present work aimed to attenuate cisplatin hepatotoxicity using nanoparticle technology and determine the prophylactic effect of an anti-inflammatory drug in diminishing this hepatotoxicity.

## Materials and methods

### Drugs and chemicals

Cetyltrimethyl-ammonium bromide (CTAB), tetraethyl orthosilicate (TEOS), castor oil was purchased from Across Co. (Germany). Cisplatin and sodium salicylate were bought from Sigma-Aldrich Chemical Company, St. Louis, MO, USA.

## Methods

### Chemical synthesis of silica nanoparticles loaded with sodium salicylate

Silica nanoparticles (Si NPs) were created as follows. An aqueous solution consisting of 1 g of CTAB in 183 ml deionized water was prepared. An oil phase solution containing 2 ml of castor oil and 15 ml of TEOS was added to the prepared aqueous solution and stirred for 5 min at 1000 rpm to obtain oil-in-water emulsion system. The above reaction mixture was sonicated at room temperature for 15 min. Following sonication, a cloudy mixture formed and was left to stand for further 24 h. The SiNPs were extracted by centrifugation at 15,000 rpm for 30 min (K241R, Centurion Scientific, UK). The SiNPs were further washed with deionized water and ethanol to remove unreacted residues. To encapsulate sodium salicylate (Sc) into the silica nanoemulsion, 2 g of (Sc) was added to the oil part of TEOS and castor oil added to aqueous phase of CTAB solution. The produced nanoparticles of Si loaded (SC) was coded as Si–Sc NPs.

As known, the aggregation particles have negative impact of the efficient role of these nanoparticles to achieve their maximum role. Characterization of the hollow structured porous silica and sodium salicylate encapsulated silica nanospheres were done using Transmission electron microscopy (TEM) & scanning electrone microscope (SEM) Detailed nano- preparation and physical characterization of the formed nanoparticles according to Shalaby et al.^26^.

### Experimental animals

All experiments were performed after approval of the protocol by the Ethical Committee of the National Research Center, Cairo, Egypt. Approval number (16/370), and the procedures were carried out in compliance with the National Institutes of Health’s Guide for the Care and Use of Laboratory Animals (NIH Publication No. 85-23, revised 1996). The design of the experiment was compatible with the ARRIVE guidelines 2.0. Sixty adult male Albino rats weighing 150–170 g, were housed at the animal facility of the National Research Center and were used in experiments after a 1-week acclimation period at 24 °C ± 1 °C in 45% ± 5% humidity with a 12 h light/12 h dark cycle. During the experimental period, a commercially balanced diet and tap water were provided ad libitum.

### Experimental design

Rats were divided randomly into five equal groups, with twelve rats in each group. Rats injected intraperitonially with either saline (control group), cisplatin (12 mg/kg BW) (cis-group), Si NPs as carrier (100 mg/kg BW/day (carrier group), or Si–Sc-NPs (100 mg/kg BW) either alone (group 4) or added along with cis (group 5). The 12 mg/kg body weight dose of cisplatin was proven to induce hepatotoxicity^27^. The duration of treatments lasted for 3 weeks pre- and 1-week post-cisplatin injection.

### Sample collection

At the end of all the treatments, rats were euthanized by decapitation. Liver tissues and blood were collected. Liver tissues were fixed in 10% neutral buffered formalin for histopathological evaluation or frozen until used for estimating liver parameters.

### Biochemical analysis

#### Preparation of liver homogenate

Immediately after excision, livers were perfused in normal saline, to remove blood cells and frozen at − 80 °C.The frozen tissues were homogenized in phosphate buffer and centrifuged for 15 min at 4000 rpm. The clear supernatant was separated and used for measuring liver functional parameters estimation^28^.

Serum alanine amino transferase (ALT), aspartate amino transferase (AST) and g-Glutamyl transferase (GGT) were determined using commercially available kits (Spectrum Company, Egypt) Serum malondialdehyde (MDA) was estimated method described previously^29^.

Nitric oxide (NO) was determined using Griess reagent, following the method described by^30^ which depends on the estimation of nitrite free radical as an indicator NO production.

The liver arylesterase activity of paraoxonase (PON1) was determined spectrophotometrically based on its ability to catalyze phenylacetate substrate. The increase in absorbance measured at 270 nm resulting due to the formation of phenol was monitored. The reaction mixture consisted of 20 mM Tris HCl buffer, pH 8.0, 1 mM CaCl_2_ and 4 mM phenyl acetate. Diluted samples (1:3) were added and the change in absorbance was noted with 20 s interval over a period of 2 min^31^.

Serum MCP1 and arginine vasopressin were measured by ELISA based assay using commercially available kits (Biovision, Inc. Beijing, China).

Tissue reduced glutathione (GSH) was determined by the method based on the reduction of Ellman’s reagent by –SH groups of GSH to form 2-nitro-smercaptobenzoic acid, the nitromercaptobenzoic acid anion which is in intense yellow color was measured by spectrophotometer.

#### Assessment of the comet assay

The comet assay was carried out according^32^ with modifications by^33^.

#### Leukocyte isolation and microgel slide preparation

Peripheral blood leukocytes were isolated by Ficoll-Paque density gradient centrifugation of whole blood (Pharmacia LKB Biotechnology, Piscataway, New Jersey, USA). Cell microgels were prepared with an initial layer of 0.7% normal melting point agarose followed by a second layer of 0.5% low melting point agarose, which was mixed with isolated leukocytes. A final layer of low-melting-point agarose was added over the second layer followed by placing coverslips, then allowed to solidify for 10 min, and then coverslips were removed.

#### Cell lysis, DNA unwinding, gel electrophoresis, and DNA staining

The slides prepared as detailed above were covered with 100 ml of fresh lysis buffer (100 mmol/l EDTA, 2.5 mol/l NaCl, 10 mmol/l Tris,1% sodium hydroxide, 1% Triton X-100, and 10% dimethyl sulfoxide, pH 10) at 4 °C for 1 h. Lysis buffer was drained from the microgels slides which were treated with DNA unwinding solution (300 mmol/l NaOH, 1 mmol/l EDTA, pH 13) for 30 min at 4 °C. Slides were subjected to electrophoresis in a DNA-unwinding buffer at 300 mA for 30 min at 4 °C. Slides were neutralized in 0.4 mol/l Trizma base solution (pH 7.5) for 10 min and were stained with ethidium bromide.

#### Visualization and analysis of comet slides

The slides were visualized (400X magnification) under fluorescence microscope (Model DM 2500, CMS GmbH; Leica Microsystems, Wetzlar, Germany). A damaged cell was characterized by its comet shaped appearance having a brightly fluorescent head and a tail to one side due to DNA strand breaks that were moved away due to electrophoresis. Percent of DNA damage was calculated by counting the damaged cells out of 100 cells per slide.

### Histopathological analysis

The formalin (10%) fixed samples were dehydrated in increasing series of ethanol, and xylene, and embedded in paraffin wax. Sections of 5 μm thickness were cut using a microtome and stained with hematoxylin and eosin (H&E) and examined under a light microscope^34^.

### Statistical Analysis

Data were analyzed by SPSS program for windows, version 25 (SPSS Inc., Chicago, IL, USA). Statistical significance was tested by one-way analysis of variance (ANOVA) followed by Bonferroni post hoc analysis. Pearson ʼs correlation was analyzed for the studied parameters. The mean difference was considered significant at the level ˂ 0.05 and highly significant at the level ˂ 0.001 Data were expressed as mean ± SE.

## Physical and biochemical characterization

### Physical characterization of nanoparticles of Si and Si loaded SC as a model drug

The particle shape of the prepared nanoemulsion of Si NPs loaded with and without Sc was investigated by transmission electron microscope (TEM). The images were captured by a JEM-2011F microscope (JEOL, Japan) at 200 kV. The hydrodynamic size of Si nanoemulsion and the nanoemulsion of Si–Sc was assesses by diluting 1 ml nanoemulsion with 10 ml of deionized water. Nanoemulsion was sonication for 10 min at room temperature. Size distributions of the nanoparticles were recorded with a Malvern Zeta sizer Nano ZS (Malvern Instruments Ltd., GB) by the DLS technique.

## Results

The utilization of silica materials as drug carriers and controlled release of drug has been extensively used since about 15 years ago. The medical applications of silica materials are desirable for their ease of formulation and biocompatibility with drugs. Encapsulation of drugs or bioactive molecules into silica nanoparticles protects them from degradation under physiological conditions, improves targeting to the affecte tissue or site, facilitates their controlled release, prolongs their time in blood circulation and minimizes adverse effects to healthy tissues. Without sono excitation in a TEOS dependent sol–gel synthesis, the hydrolysis of TEOS requires either base or acid base catalysts. The hydrolysis of TEOS is relatively slow as compared faster silica condensation reaction at neutral pH. However, TEOS could be hydrolyzed by sonochemical excitation. Ultrasonic irradiation, used in place of widely employed basic or acidic catalyst was used for production of acoustical cavitation within the liquid H_2_O/TEOS reactants.

**Figure 1:**
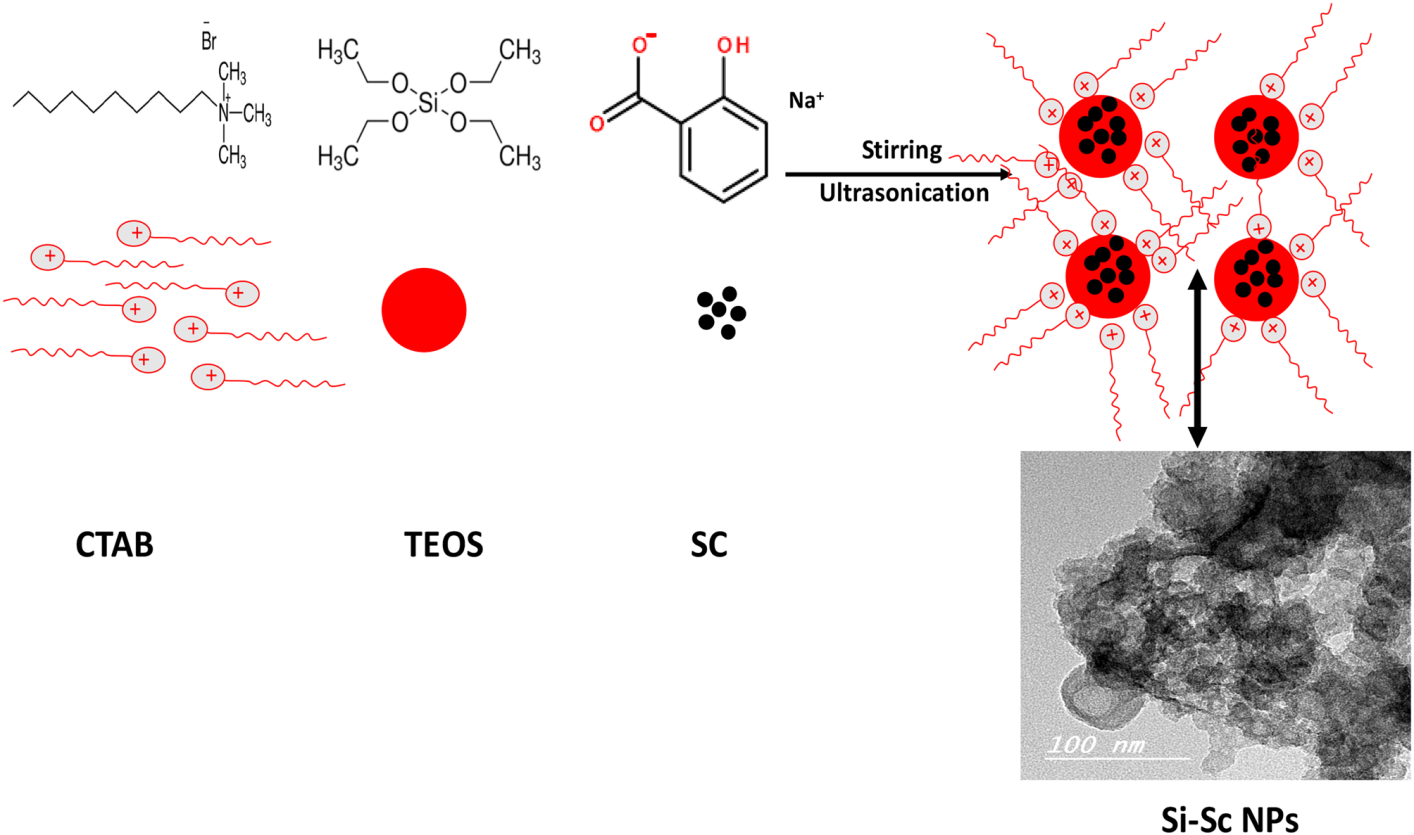
Shows that sodium salicylate is loaded to TBOS that was introduced into the preformed porous silica then transformed in aqueous CTAB solution to stabilize the form.

Figure 1 describes the steps for the formation of hollow structured porous silica encapsulated with (Sc) as a model drug. The nanoemulsion was successfully prepared using TEOS, CTAB and (Sc) as precursor for silica, surfactant and model drug respectively.

### Characterization of silica and silica loaded sodium salicylate

**Figure 2:**
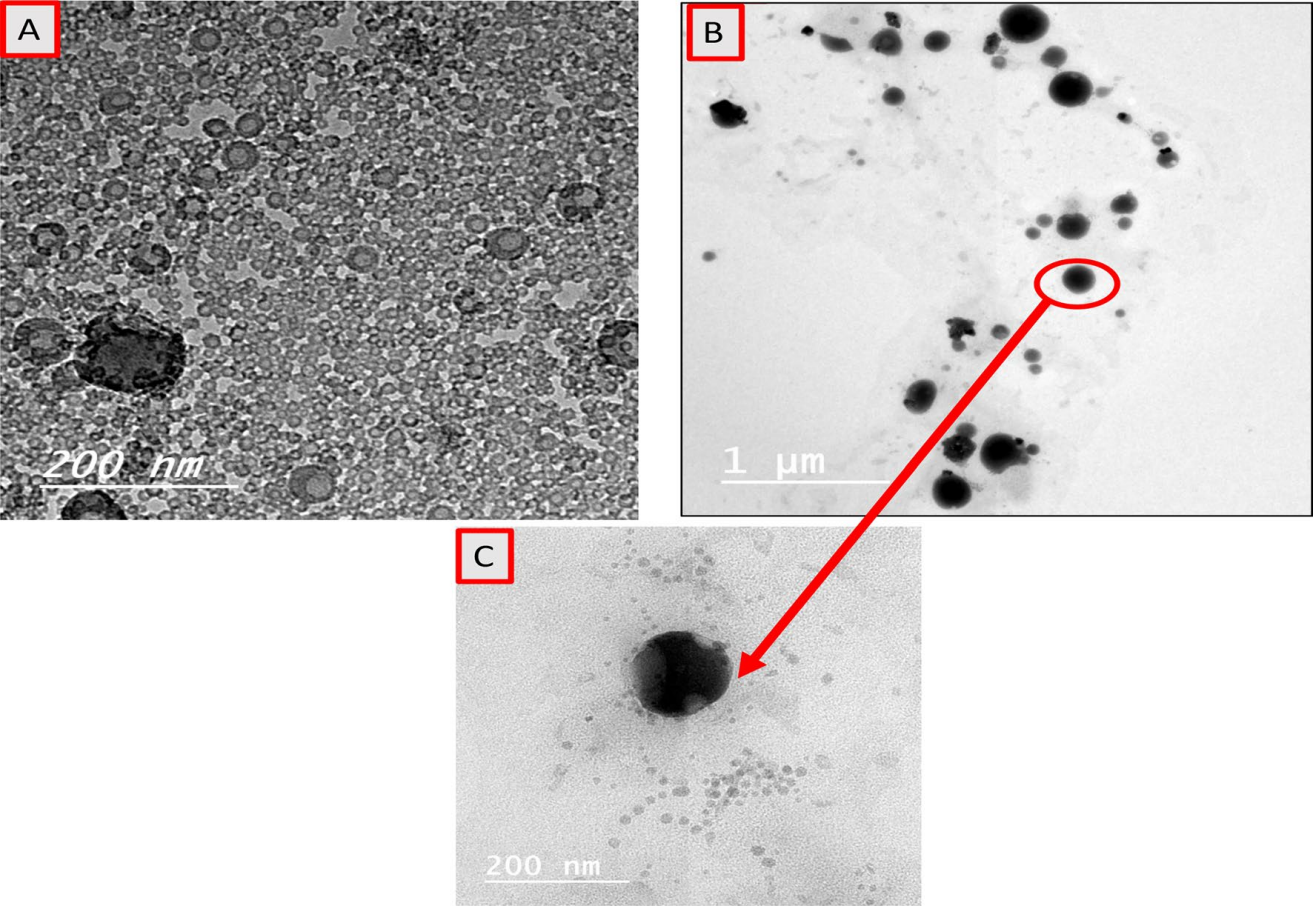
TEM images showing (**A**) a hollow and nested sphere within a sphere interior of different contrast of silica emulsion however, (**B**) shows that salicylate had been loaded to silica nano emulsions then was stabilized by CTAB to keep the characterization of nanoparticles as shown in (**C**).

Figure 2 represents the TEM images of silica nanoemulsion and nanoemulsion of silica encapsulated with (Sc). It can be clearly seen (Fig. 2A) that the silica nanoemulsions are hollow, contain a nested sphere-within- a sphere interior of different contrasts. On the other hand, Fig. 2B demonstrate the particle shape of silica nanoemulsion loaded with (Sc). It is clearly seen that the particle shape significantly changed when compared with the hollow structure of silica nanoparticles. It is observed in Fig. 2B that there is dark middle region due to the encapsulation of (Sc). In addition, Sc occupied the internal space of the inner hollow spheres as evident from high magnification TEM figure (Fig. 2C).

By and large, all particles of silica and silica loaded with the model drug (Sc) is still in the form of nanometre size. Due to encapsulation of salicylate, a marginally increased size could be seen.

The addition of CTAB plays a crucial for stabilizing synthesized silica nanoemulsion with and without (Sc). In the presence of CTAB, some well-spaced hollow silica nanospheres are observed due presence of positive charges. It is also observed that the solubility of CTAB in the encapsulated oil phase is high in the presence of castor oil. The ultrasound-induced silica helps in the rapidly formation of homogeneity and well stabilized nanoemulsion.

**Figure 3:**
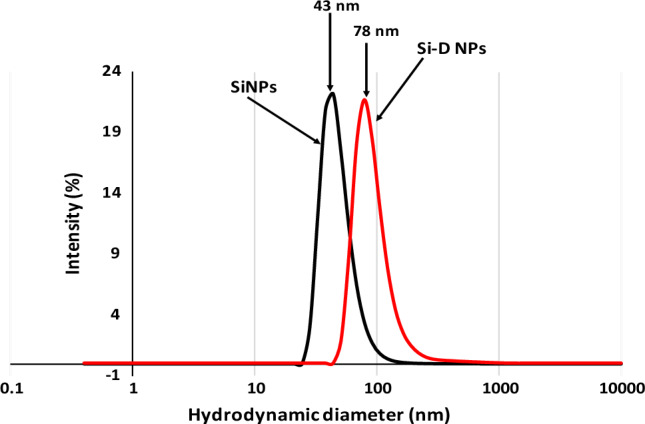
Hydrodynamic size of nano emulsion of silica nanospheres as represented in black peak with size 43 nm and silica nanospheres encapsulated with sodium salicylate as represented in red peak with size 78 nm (in the accepted nanoparticle size).

Dynamic light scattering (DLS) results showed that Si nanosphere have a mean hydrodynamic diameter of 43 nm in water, showing that Si nanosphereis are non-aggregating. After encapsulation with (Sc), the average hydrodynamic size increases to 78 nm. This indicates the negative charges on silicate were not higher in magnitude to nullify the positive charge of CTAB, and that has the capability to repulsion the silica particles from each other. The DLS analysis data (Fig. 3) is consistent with TEM images (Fig. 2).

Data of the studied parameters are represented as mean ± SE. Levels of AST, ALT and δGT were highly statistically significant increase in rats injected by cis when with the control group. Meanwhile, a statistically significant reduction has been achieved when previously injected with sodium salicylate nanoparticles (Si–Sc-NPs). However, the levels of AST, ALT and δGT have been marginally changed in groups injected with carrier or Si–Sc-NPs in relation with the control group, indicating the supplemented nanoparticles safety as represented in (Table 1).

**Table 1:** Serum markers in the studied groups.

	Control	Carrier	Cisplatin	Sod.sal	Sod.sal + cis
AST (U/L)	78.5 ± 5.6^c^**^,e^**	86.2 ± 5.5^c^**^,e^*	125 ± .97^a^**^,b^**^,d^**^,e^**	73.3 ± 4.1^c^**^,e^**	104 ± 1.3^a^**^,b^**^,c^**^,d^**
ALT(U/L)	32.7 ± 1.22^c^**^,e^**	31.9 ± 1.63^c^**^,e^**	74.6 ± 1.62^a^**^,b^**^,d^**^,e^**	28.1 ± 1.64^c^**^,e^**	52.9 ± 1.61^a^**^,b^**^,c^**^,d^**
GGT(U/L)	18.8 ± .96^c^**^,e^**	21.92 ± 1.01^c^**^,e^**	49.6 ± 1.92^a^**^,b^**^,d^**^,e^**	19.7 ± 1.15^c^**^,e^**	32.4 ± 1.64^a^**^,b^**^,c^**^,d^**
MCP1 (Pg/ml)	340.7 ± 7.68^b^**^,c^**^,e^**	394.3 ± 6.81^a^**^,c^**^,e^**	661.3 ± 8.51^a^**^,b^**^,d^**^,e^**	352.7 ± 7.42^a^*^,b^**^,c^**^,e^**	485.7 ± 8.72^a^*^,b^**^,c^**^,d^**
AVP (Pg/ml)	10.7 ± .04^c^**^,e^**	9.8 ± .02^c^**^,e^**	4.7 ± .31^a^**^,b^**^,d^**^,e^**	10.2 ± .09^c^**^,e^**	7.5 ± .19^a^**^,c^**^,d^**

Data are presented as mean ± SE.

*Significance ˂ 0.05 and **highly significant ˂ 0.001.

^a^Significantly different when compared to control group.

^b^Significantly different in relation to to carrier group.

^c^Is the significantly different when compared to cisplatin group.

^d^Is the significantly different when matched to sodium salicylate group.

^e^Is the significantly different when compared to the group treated with sodium salicylate followed by cisplatin.

Oxidant and antioxidant parameters of oxidative stress as well as inflammatory marker are shown in Tables 1 and 2. Liver MDA as well as NO levels were found to be significantly enhanced, but PON 1 was significantly truncated in cis group as compared to control group, leading to augmented oxidative stress.

**Table 2:** Tissue markers and peripheral blood DNA damage in the studied groups.

	Control	Carrier	Cisplatin	Sod.sal	Sod.sal + cis
MDA (µmol/g)	94.9 ± 2.26^b^**^,c^**^,e^**	113.8 ± 1.38^c^**^,e^**	243.3 ± 3.92^a^**^,b^**^, d^**^,e^**	101.6 ± 2.89^c^**^,e^**	194.2 ± 2.28^a^**^,b^**^,c^**^,d^**
NO (µmol/g)	15.7 ± .24^c^**^,e^**	16.2 ± .33^c^**^,e^**	43.4 ± .72^a^**^,b^**^,d^**^,e^**	17.3 ± .25^c^**^,e^**	34.2 ± 1.76^a^**^,b^**^,c^**^,d^**
PON (µmol/g)	95.6 ± 2.62^c^**^,e^**	94.2 ± 2.94^c^**^,e^**	53.2 ± 2.83^a^**^,b^**^,d^**^,e^**	94.3 ± 1.12^c^**^,e^**	64.2 ± 2.26^a^**^,b^**^,d^**
GSH (µmol/g)	10.2 ± .21^c^**^,e^**	10.4 ± 1.89^c^**^,e^**	5.7 ± .12^a^**^,b^**^,d^**^,e^**	10.6 ± .25^c^**^,e^**	8.1 ± .13^a^**^,b^**^,c^**^,d^**
DNA (%)	7.5 ± .93^c^**^,e^**	8.1 ± .71^c^**^,e^**	59.1 ± 2.52^a^**^,b^**^,d^**^,e^**	9.1 ± .39^c^**^,e^**	32.8 ± .88^a^**^,b^**^,c^**^,d^**

Data are presented as mean ± SE.

** highly significant ˂ 0.001.

^a^Is the significantly different when matched to control group.

^b^Is the significantly different when compared to carrier group.

^c^Is the significantly different when compared to cisplatin group.

^d^Is the significantly different when compared to sodium salicylate group.

^e^Is the significantly different when compared to the group treated with sodium salicylate followed by cisplatin.

Moreover, these results were improved in Si–Sc NPs protected groups in comparing with cis group, which indicate the antioxidant capacity of those formulated supplemented agent.

Pearson correlation was among the studied groups as shown in (Table 3). Levels of liver reduced glutathione were negatively correlated with both the percentage of leucocytes DNA damage and serum arginine vasopressin levels showing a highly statistically significant correlation; − 0.938 and − 0.909 respectively. However, there was a highly statistically positive correlation between the percent leucocytes DNA damage and serum arginine vasopressin levels (0.958).

**Table 3:** Pearson ʼs correlation coefficient of the studied parameters.

	AST	ALT	GGT	L.MDA	L.NO	L.PON	S.MCP1	DNA	L.GSH	S.AVP
AST	–	0.772**	0.726**	0.797**	0.779**	− 0.742**	0.809**	0.359*	− 0.280*	− 0.344*
ALT		–	0.881**	0.925**	0.949**	− 0.880**	0.856**	0.950**	− 0.916**	− 0.908**
GGT			–	0.891**	0.913**	− 0.775**	0.838**	0.911**	− 0.840**	− 0.860**
L.MDA				–	0.931**	− 0.910**	0.909**	0.971**	− 0.914**	0.944**
L.NO					–	− 0.826**	0.901**	0.956**	− 0.906**	0.920**
L.PON						–	− 0.828**	− .896**	0.865**	0.860**
S.MCP1							–	0.918**	− 0.915**	0.919**

Data are presented as (r) correlation coefficient, * significance ˂ 0.05 and ** highly significant ˂ 0.001.

### Leucocytes DNA damage assessments using comet assays.

**Figure 4:**
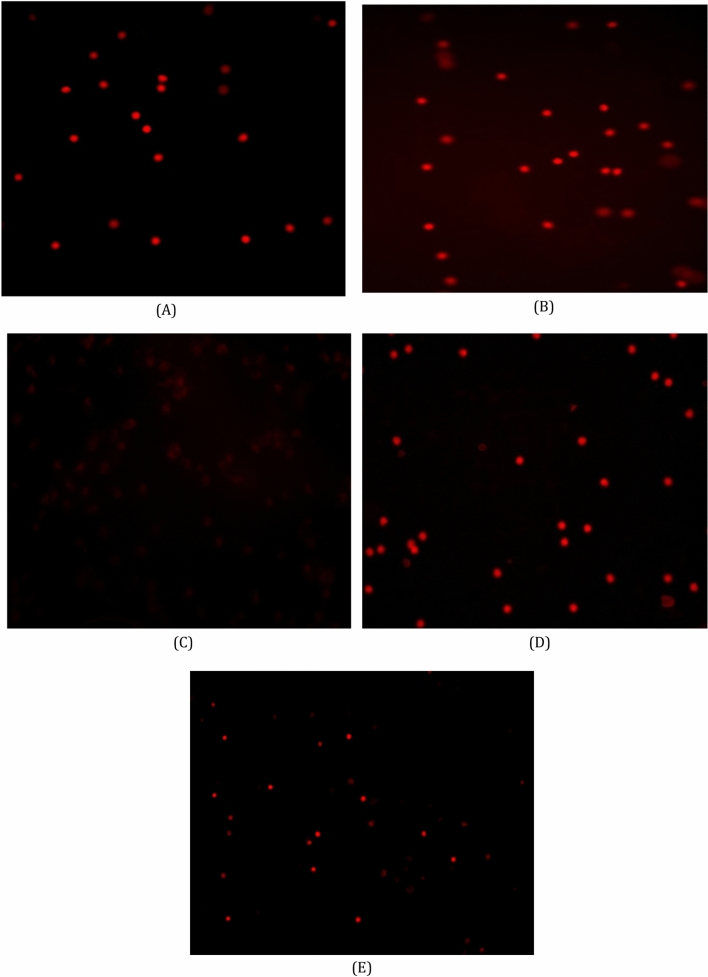
Photomicrograph of comet assay for detection of DNA damage showing nuclei of leucocytes of blood.

leucocytes DNA damage assessments using comet assays are shown in the different studied groups as shown (Fig. 4A–E); (A) Photomicrograph of comet assay for normal rats showed intact and bright DNA, (B) DNA damage in leucocytes of carrier group showing an intact and bright DNA, (C) Photomicrograph of comet assay for cisplatin-treated rats, showing a highly damaged DNA due to the migration of damaged DNA fragments throughout electrophoresis leading to a shadowy look of DNA, (D) DNA damage in leucocytes of sodium salicylate group showing an intact and bright DNA, (E) Photomicrograph of comet assay for sodium salicylate + cisplatin-treated rats, showing a slightly modulated damaged DNA due to the migration of damaged DNA fragments throughout electrophoresis leading to a faint look.

### Histopathological results

**Figure 5:**
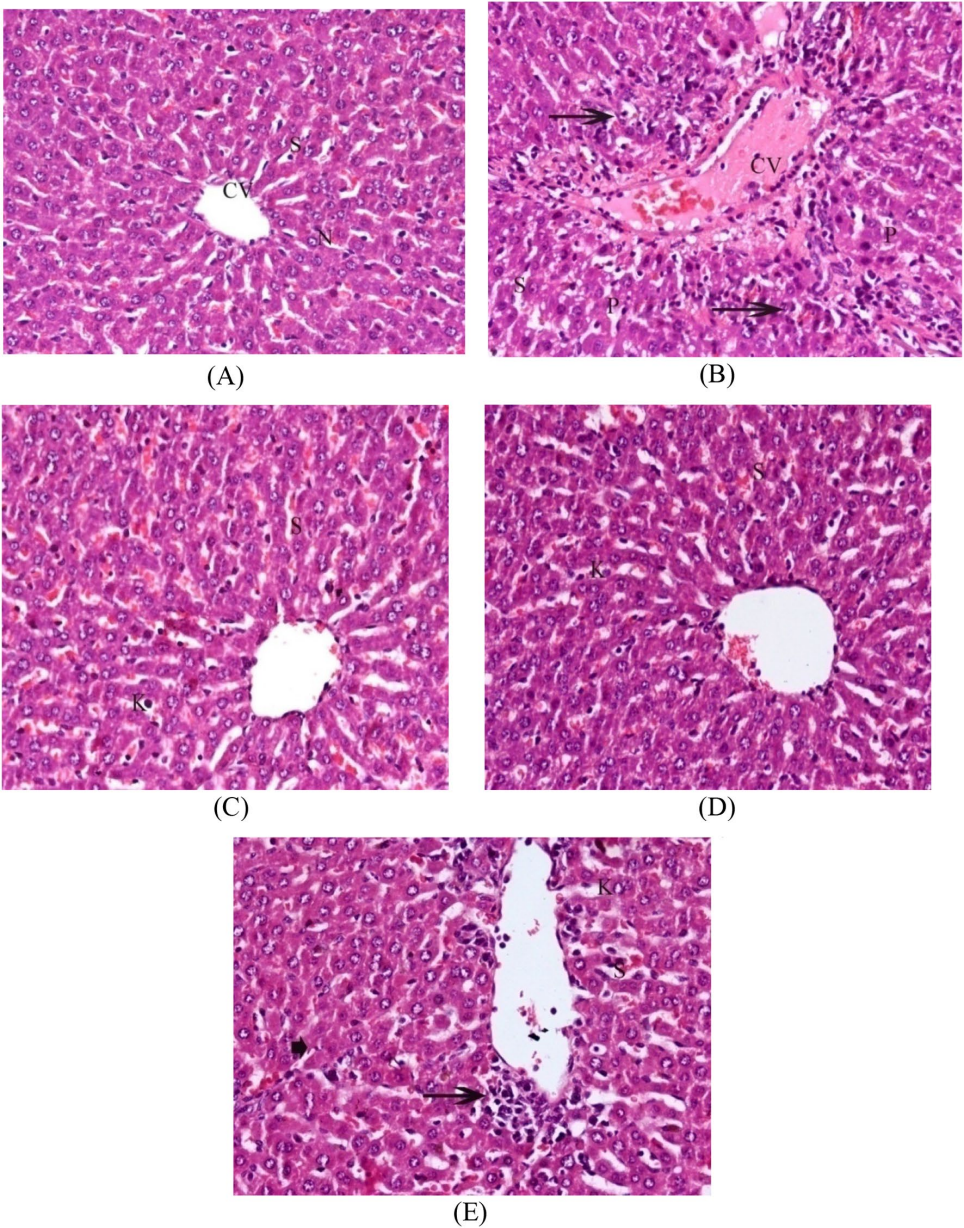
Histological examinations of liver tissue sections; (**A**) Liver section of control rats exhibiting normal structure of liver, central vein (CV), prominent nucleus (N) separated with blood sinusoids (S) (H & E X 400), (**B**) Photomicrograph of liver sections of cisplatin group showing severe hepatic necrosis associated with mononuclear cells infiltration around central vein (thin arrow), congestion central vein (CV), hemorrhage blood sinusoids (S).and pyknotic nuclei (P) (H & E, 400X), (**C**) Liver section of Carrier group exhibiting normal histology of liver, with few Kupffer cells (K) and hemorrhage blood sinusoids (S) (H & E, 400X), (**D**) liver section of Sodium salicylate group showing few Kupffer cells (K) and blood sinusoids(S) (H & E, 400X), (**E**) Photomicrograph of section from liver of Sodium salicylate + Cis group showing inflammatory cells (thin arrow), necrotic cells (arrowhead), Kupffer cells (K) congestion central vein (CV) and blood sinusoids(S) (H & E X 400).

Normal hepatocyte histology, central vein, sinusoidal spaces and prominent nucleus were seen in the liver sections from control rats (Fig. 5A). In contrast, rats exposed to Cis demonstrated a marked hepatocyte damage characterized by cytoplasmic vacuolation, degeneration, and centrilobular necrosis associated with mononuclear cells infiltration around central vein, which was congested and enlarged. The sinusoid walls showed numerous Kupffer cells, hemorrhages with sinusoids dilatation, and nuclei were pyknotic showing condensed chromatin (Fig. 5B). In the group received carrier only showed nearly normal structure with few Kupffer cells and hemorrhage blood sinusoids (Fig. 5C). Histopathological examination of the group received Si–Sc-NPs showed the hepatic structure appear almost normal except for a few Kupffer cells and blood sinusoids (Fig. 5D). This effect was ameliorated when treated with Si–Sc-NPs and cis in inflammatory cells, necrotic cells and congestion blood sinusoids and central vein (Fig. 5E).

## Discussion

Generally, in a clinical setting, cisplatin is given to cancer patients as a pretreatment to determine its tolerance in advance. Coinciding with this the rats in the present study were pretreated with cisplatin.

Our findings revealed that the cisplatin was able to induce acute hepatotoxicity in rats. Also, silica nanoparticles loaded sodium salicylates (Si–Sc-NPs) protected the liver from damage induced by cis. These findings were backed up by reduced plasma AST, ALT as well as GGT with the obvious normal liver histology. In our study, compared to control, increased levels of serum AST, ALT and GGT levels in cis-treated rats indicates the capacity of cisplatin to induce hepatotoxicity. The data was consistent with previous studies wherein similar increase in liver functional markers consequent to leakage from the damaged liver was reported in response to cisplatin treatment indicating hepatotoxicity, hepatic dysfunction and liver damage^35^. Several studies have also reported a significant elevation in serum SGPT and SGOT levels in response to cisplatin^36,37^.

Cisplatin‐induced liver damage is correlates well with the increased lipid peroxidation (LPO) and depletion of reduced glutathione as result of ROS generation and altered antioxidant enzyme activities. Cisplatin has been shown to attenuate mitochondrial dysfunction, electron transfer system, leading to enhanced superoxide anions, H_2_O_2_, and hydroxyl radical production eventually causing mitochondrial dysfunction^38^. MDA is an important biomarker in determining oxidative stress and cellular damage as it is an end product of lipid peroxidation^39,40^.

Most cellular proteins are susceptible to oxidative stress by two pathophysiological events including peroxy nitrite modification and lipid peroxidation^41,42^. In the current study, obvious increases in liver MDA levels as well as NO were observed accompanying with a decrease in PON 1 contents in the cis group. The increase of MDA due to increases in lipid peroxidation and oxidative stress is a reasonable explanation for liver injury induced by cis in the following study. Moreover, many studies reported that increases in oxidative stress and ROS signaling cascade were linked to hepatic stellate cells, which also produce ROS^43^. Besides ROS, NO represents other active radical synthesized by nitric oxide synthase (NOS). Here we demonstrated that pretreatment of rats with sodium salicylates significantly attenuated both lipid peroxidation and cisplatin-induced protein nitration. These data are in line with that of^44^.

Exposure of rats to cisplatin significantly induced MDA formation in the liver; however, protection by salicylates significantly decreased MDA levels. Similarly, a decrease in liver tissue GSH levels was observed when compared to control as well as cisplatin alone group. Earlier reports have also reported a significant reduction in tissue GSH levels upon cisplatin administration^16,45^.

Zeng et al. developed a novel proteomics strategy combining functionalized magnetic nanoparticle affinity probes with mass spectrometry to capture and identify proteins specifically responding to the major DNA lesion caused by cisplatin that induces apoptosis. They concluded that it could promote understanding cellular response to cisplatin- damaged DNA and motivate further precise explanation of cisplatin mechanism of action^46^.

In this current study we found that MDA levels were significantly augmented in cisplatin group as compared to control. These findings are in line with earlier studies wherein cisplatin treatment led to a significant increase in MDA levels in rat liver^47,48^.

In our study, administration of sodium salicylates statistically decreased MDA levels when compared with cisplatin administration. These data are consistent with previous studies describing the effects of on MDA in liver and kidney^49^. Also, our results revealed a statistically significant elevation in nitric oxide level in liver tissue of cisplatin treated rats. Nitric oxide, a highly reactive molecule, is synthesized in liver by parenchymal and non-parenchymal cells from L-arginine catalyzed by inducible nitric oxide synthase^50^. Consistent with our observations, liver NO has been shown to be elevated in response to cisplatin treatment^23,51^.

In our study, administration of sodium salicylates significantly reduced NOx level in cisplatin-induced rats. This result is consistent with those reported by Carnovale et al.^52^. All these findings of oxidative stress induced by cisplatin were further supported by the percentage of DNA damage evaluated by the comet assay in peripheral blood leucocytes. Zeng et al. have demonstrated findings that help understand the cellular response to cisplatin induced DNA damage and elucidation of the mode of action of cisplatin^46^.

Our results showed significant change in the levels of antioxidant markers, GSH and PON in rats injected with cisplatin. Sodium salicylates administration has restored antioxidant status that approaches control levels. This result correlates with that of Goyal et al.^45^.

Non-enzymatic antioxidant, GSH was significantly downregulated in liver tissues of cisplatin administered rats as against that in control. Sodium salicylate treatment however led to a marked increase in liver GSH indicating the antioxidant nature of this compound. Similar observations were reported earlier where sodium salicylate was shown to act as antioxidant^23,51^.

Our revealed a statistically highly significant increase in MCP-1 in cisplatin injected group when compared to their normal control counterparts and this was improved when previously treated with sodium salicylates loaded nanoparticles. Macrophage activation, pro-inflammatory responses, macrophage activation and hepatic steatosis are regulated by the liver in liver disease. Previous studies have suggested an association between inflammatory cell activation and fatty acid metabolic pathways in liver injury and their likely participation in disease progression. Presumably, pharmacologically blocking MCP-1 in liver may prove to be beneficial due to the inhibition of inflammatory pathways that contribute to disease pathology^53,54^.

Our results revealed an arginine vasopressin deficiency in cisplatin induced liver toxicity. This result is in line with that of Wagener et al.^55^.

In conclusion, drug hepatotoxicity indicated by elevated levels of AST, ALT and GGT in Cis–induced hepatotoxicity rats associated with disturbances in oxidative stress parameters were augmented by protective (Si–Sc-NPs). Findings of increased MDA and NOx levels underscore the participation of nitric oxide in promoting cisplatin-induced liver toxicity. Also, this study indicates that relative deficiency of vasopressin and increased sensitivity of vasopressin may contribute to systemic vasodilation in liver disease. These findings may have potential implications in attenuating drug induced hepatotoxicity.

## Data Availability

The data used during the current study are available from the corresponding author on reasonable request.
